# A clue on bee glue: New insight into the sources and factors driving resin intake in honeybees (*Apis mellifera*)

**DOI:** 10.1371/journal.pone.0210594

**Published:** 2019-02-06

**Authors:** Nora Drescher, Alexandra-Maria Klein, Thomas Schmitt, Sara Diana Leonhardt

**Affiliations:** 1 Institute of Ecology, Leuphana University of Lüneburg, Lüneburg, Germany; 2 Chair of Nature Conservation and Landscape Ecology, University of Freiburg, Freiburg, Germany; 3 Department of Animal Ecology and Tropical Biology, University of Würzburg, Biocenter—Am Hubland, Würzburg, Germany; College of Agricultural Sciences, UNITED STATES

## Abstract

Honeybees (*Apis mellifera*) are threatened by numerous pathogens and parasites. To prevent infections they apply cooperative behavioral defenses, such as allo-grooming and hygiene, or they use antimicrobial plant resin. Resin is a chemically complex and highly variable mixture of many bioactive compounds. Bees collect the sticky material from different plant species and use it for nest construction and protection. Despite its importance for colony health, comparatively little is known about the precise origins and variability in resin spectra collected by honeybees. To identify the botanical resin sources of *A*. *mellifera* in Western Europe we chemically compared resin loads of individual foragers and tree resins. We further examined the resin intake of 25 colonies from five different apiaries to assess the effect of location on variation in the spectra of collected resin. Across all colonies and apiaries, seven distinct resin types were categorized according to their color and chemical composition. Matches between bee-collected resin and tree resin indicated that bees used poplar (*Populus balsamifera*, *P*. *x canadensis*), birch (*Betula alba*), horse chestnut (*Aesculus hippocastanum*) and coniferous trees (either *Picea abies* or *Pinus sylvestris*) as resin sources. Our data reveal that honeybees collect a comparatively broad and variable spectrum of resin sources, thus assuring protection against a variety of antagonists sensitive to different resins and/or compounds. We further unravel distinct preferences for specific resins and resin chemotypes, indicating that honeybees selectively search for bioactive resin compounds.

## Introduction

Honeybees (*Apis mellifera*) are threatened by numerous pests and pathogens [1–3]. They further have to deal with multiple environmental stressors caused by agricultural intensification, such as pesticides, alteration of foraging landscapes, reduced resource diversity and rapidly spreading new pests and pathogens [4–9]. Impacts of single or combined stressors can have devastating consequences for honeybee health and can even lead to the total collapse of colonies as seen e.g. in the high winter losses in Europe and North America [10]. To increase the resilience of colonies, instead of only addressing the symptoms, a sustainable strategy is to promote the bees’ natural defenses [11–14]. Honeybees perform several collaborative behaviors, such as allo-grooming, nest hygiene or the collection and use of antimicrobial plant resins, which are referred to as social or external immunity and play an essential role for colony health [15–18]. While much attention has been paid to hygienic behaviors, the use and role of plant resins has been largely neglected until recently [19,20].

Plant resin is a sticky, water insoluble substance which plants secrete primarily to protect injured tissue, young sprouts or leaf buds from herbivore and/or pathogen attack [21]. Resin comprises a chemically complex mixture of often more than 300 substances (mostly phenolic compounds such as flavonoids, aromatic carboxylic acids, and benzopyranes, and terpenoids), many of which show antimicrobial and/or repellent properties [21]. The chemical composition of resin is typically plant species-specific, but can vary greatly—both qualitatively and quantitatively—within and between plant families and even among closely related plant species [21].

*Apis mellifera* as well as some tropical stingless bees (Apidae: Meliponini) use resins for nests construction and as defense against pests and pathogens [19,22–24]. Resin is often mixed with various amounts of wax, resulting in a tough, sticky matter which is called propolis or bee glue [25]. Non-managed, feral colonies of honeybees coat their entire nest interior with a thin propolis layer referred to as “propolis envelope” [26]. Such a “propolis envelope” can reduce the load of microbes and decrease the expression of (at least two) immune-related genes [19]. Moreover, several studies demonstrated that propolis reduced the growth of important microbial bee pathogens, i.e. *Paenibacillus larvae*, the causative agent of American Foulbrood [27–29], and *Ascophaera apis*, a fungal parasite that causes chalkbrood [28,30]. Resin/propolis further enhanced the immune activity of individual bees, which enabled them to more effectively fight pathogenic challenges [19,31–34]. Consequently, resin plays a crucial role for colony health and represents an essential resource for honeybees.

Despite its obvious importance, we still know relatively little about resin collection in honeybees. While tropical stingless bees were shown to collect resins from a wide range of plant species [23,35], precise information on the plant sources used by *Apis mellifera* is still scarce and largely restricted to sporadic observations (e.g. of foragers on hybrid *Populus* spp., France [36], and coniferous trees, North America [37]), few chemical comparisons (e.g. for *Baccharis dracunculifolia*, Brasil [38], and *Populus deltoides*, *P*. *balsamifera* and hybrid *Populus* spp., North America [39,40]) and inferences from major chemical compounds of propolis [41–43]. However, propolis is typically produced by mixing different resins (and wax), which renders inferences on single original resin sources or on variation among different resin sources difficult, as some compounds (e.g. camphene, *alpha*- and *beta*-pinene, limonene and myrcene) occur in resins of numerous plant taxa [21]. Moreover, identifying botanical sources based on observations is challenging, because the actual foraging process is difficult to observe as it is usually performed by a relatively low number of bees [30] and as it can occur high up in the trees [44].

Based on the above listed studies, poplar species (hybrid *Populus* spp.), horse chestnut (*Aesculus hippocastanum*), alder (*Alnus* spp.), birch (*Betula* spp.), willow (*Salix* spp.) and some others are currently considered the main resin sources for honeybees in temperate regions [25,43,45]. However, most plant species have not yet been confirmed as actual resin source through thorough chemical comparison (but see [39]), and information on possible preferences as well as variation in the collection of different resin sources over space and time is largely missing.

Such information can only be inferred by tracing the collection behavior of single resin foragers, e.g. by collecting and chemically analyzing resin loads from hind legs of returning foragers. To the best of our knowledge, there are only two studies from North America that chemically analyzed loads from individual resin foragers [39,40]. However, both studies investigated resin collection of bees for only one apiary/site and do therefore not provide information on variation between sites and over time.

Following up on the pioneering results of [39], our study aimed to identify botanical sources of resins collected by *Apis mellifera* in Western Europe through comparative chemical analysis at the level of individual foragers. We further examined the resin intake of 28 different colonies from seven different apiaries placed at locations differing in the surrounding landscape structure and thus composition and/or relative abundance of tree species to assess the effect of location on variation in the collected resin spectra.

## Material & methods

### Study sites and honeybee colonies

Observations of resin collection behavior and sampling of resin from bees and trees were conducted at seven different sites in Lower Saxony, in northwestern Germany, between June and October 2012/2013. Distances between all sites ranged from minimum 1.3 km to maximum 38 km and, for sites included in the analysis of site specific differences, from 4.6 km to 29.5 km, respectively (S1 Fig). The habitat surrounding apiaries was characterized by either an agricultural landscape with croplands (dominated by corn maize, rapeseed and potato), pastures and mixed forests (N = 5 sites) or an urban landscape with parks, small gardens or allotments and roadside trees (N = 2 sites; Table 1). In general, this region is dominated by a continental climate with typical forest assemblages comprising oak (*Quercus robur /petraea*), beech (*Fagus sylvatica*), pine (*Pinus sylvestris*) and spruce (*Picea abies*). Other common occurring deciduous tree species belong to the genera birch (*Betula)*, poplar (*Populus*), alder (*Alnus*), maple (*Acer*), ash (*Fraxinus*) and linden (*Tilia*). Our study region thus comprised several tree species assumed to be resin sources of *Apis mellifera*, i.e. *Betula spp*., *Aesculus hippocastanum*, *Alnus spp*. and several species within the genus *Populus* (i.e. several unknown hybrids of *P*. *xcanadensi* of the section *Aigeiros*, *P*. *balsamifera* of the section *Tacamahaca*, and *P*. *tremula* of the section *Populus*). Individuals from all species were available within the flight range of each apiary, except for *P*. *balsamifera* which occurred only at two sites and *A*. *hippocastanum* which was missing at one site.

**Table 1:** Overview of the study sites and honeybee colonies.

**Site**^**a**^	**Colony ID**	**N of observations**	**N of resin foragers**^b^	**Propolis sample**	**Environment**^c^
Bb	1	1	5		agricultural (mixed forest, pastures)
Bb	2	2	18		agricultural (mixed forest, pastures)
Bb	3	1	3		agricultural (mixed forest, pastures)
Bb	4	1	5		agricultural (mixed forest, pastures)
Bb	5	2	19		agricultural (mixed forest, pastures)
Et	1	1	5		agricultural (coniferous forest)
Et	5	1	5		agricultural (coniferous forest)
Ez	1	1	7	x	agricultural (mixed forest, pastures)
Ez	2	1	19		agricultural (mixed forest, pastures)
Gr	1	2	4		agricultural (cropland, patches of mixed forest)
Gr	2	3	27		agricultural (cropland, patches of mixed forest)
Gr	3	6	49		agricultural (cropland, patches of mixed forest)
Gr	5	3	11		agricultural (cropland, patches of mixed forest)
Gr	7	6	48	x	agricultural (cropland, patches of mixed forest)
Gr	9	2	29	x	agricultural (cropland, patches of mixed forest)
Lg	4	8	15		urban (gardens, small forest)
Lg	5	11	43		urban (gardens, small forest)
Lg	6	12	36		urban (gardens, small forest)
Lg	11	10	24	x	urban (gardens, small forest)
Lg	12	9	22	x	urban (gardens, small forest)
Ml	2	4	34	x	urban (gardens, small forest)
Ml	4	3	23		urban (gardens, small forest)
Ol	1	4	21		agricultural (pastures, groves)
Ol	2	5	25		agricultural (pastures, groves)
Ol	3	2	17		agricultural (pastures, groves)
Ol	7	5	24		agricultural (pastures, groves)
Ol	8	6	18		agricultural (pastures, groves)
Ol	9	8	88		agricultural (pastures, groves)

^a)^ Different sites are described by abbreviations (i.e. “Bb” Bienenbüttel, “Et” Ebstorf, “Ez” Eitzen, “Gr” Grünewald, “Lg” Lüneburg, “Ml” Melbeck, “Ol” Oldershausen).

^b)^ Total number of resin collecting bees observed per colony.

^c)^ Apiaries were located either in agricultural or urban environments. The surrounding environment differed between apiaries (described in brackets).

For each colony (colony ID) number (N) of observation days, total number (N) of observed resin foragers and whether (x) or not (no entry) propolis samples were taken is given. In total, resin intake was observed in 28 *Apis mellifera* colonies of seven different apiaries between 12 and 6 pm.

All land owners and bee keepers gave permission for sampling where necessary. Resin samples of trees where obtained from trees that were located at public sites where no specific permission was required.

### Observation of resin intake

**Fig 1:**
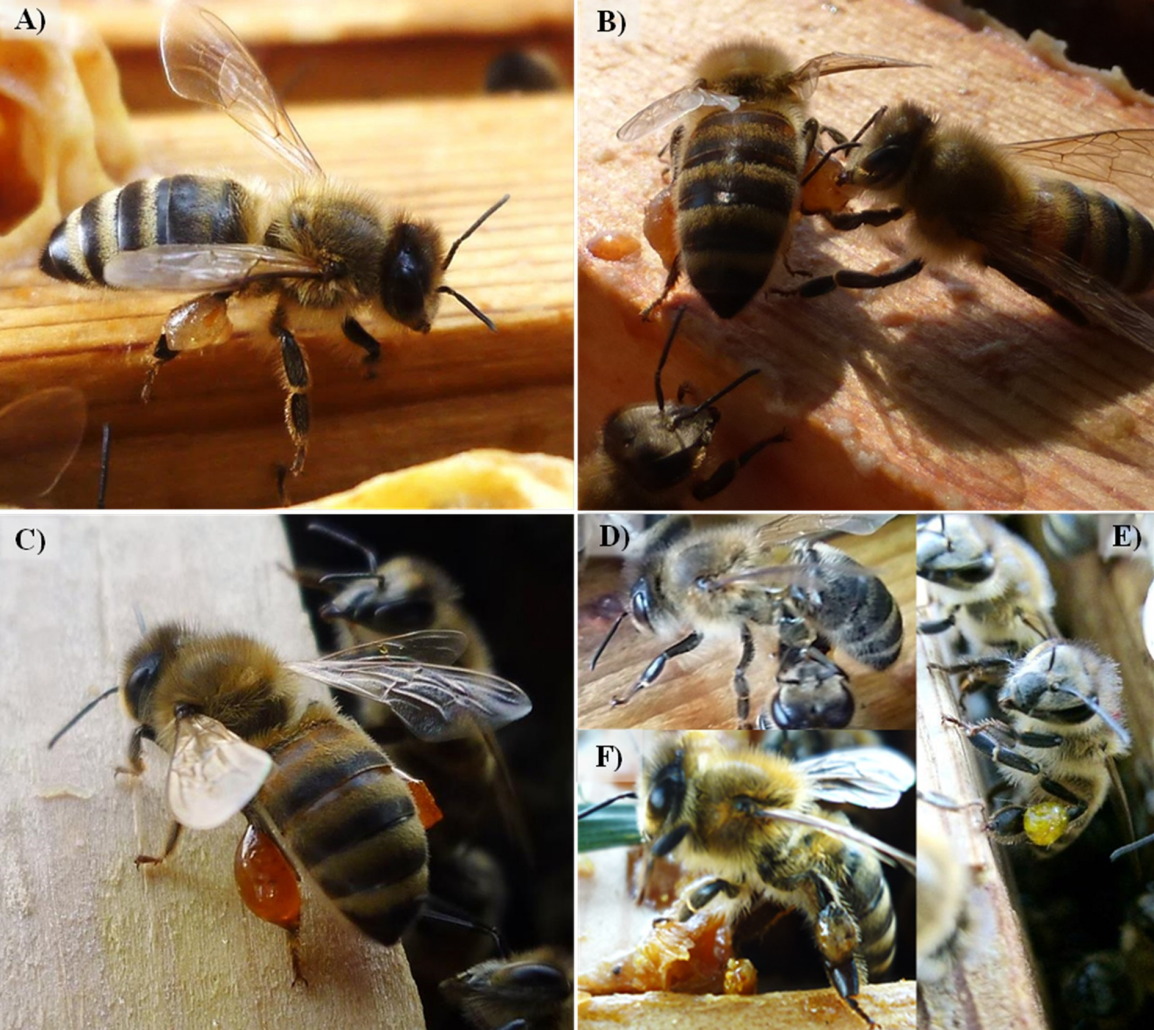
Resin foragers carrying different resin types on their hind legs. Different resin (color-) types (A-F) were used for visual determination of different resin types: A) “ocher”, B-C)”orange”, D) “clear”, E) “yellow”, F) “brown”.

Resin intake of 28 *A*. *mellifera* colonies of seven different apiaries was observed by opening each bee colony and searching for resin foragers on the top of the frames for 15 minutes. Because unloading of resin loads frequently occurs on the top of the frames with the help of one or two other bees (personal observation) here, resin foragers can be easily identified and captured. Different resins collected by bees were categorized according to their color [46], resulting in seven distinct resin color types, i.e. “orange”, “red”, “ocher”, “green-brown”, “light clear”, “yellow” and “whitish” (all also including varieties of the respective color, e.g. light-ocher or dark-orange, see Fig 1). Categories were then confirmed chemically (see below). Data from all 28 colonies was used to capture the whole spectrum of resins collected by bees in the study area. All resin color types were further sampled for chemical analysis as described below. To avoid multiple counts of the same bee individual, resin foragers were either kept in plastic tubes until the end of the observation or their resin loads were taken before releasing them again. Colonies were observed three to eight times (16 colonies) or, due to time constrains, only one to two times (12 colonies).

To assess site-specific differences in the spectra and diversity of resins gathered by colonies we further recorded the number of returning resin foragers carrying a particular color type within time intervals of 15 minutes. Analysis of site-specific differences was restricted to colonies that were observed at three or more different days (Table 1). Each of these 16 colonies was observed at 6 ± 3 (mean ± standard deviation (SD)) days between 12 and 6 pm (Table 1). Finally, temperature was recorded for each observation interval to assess possible influences on the resin intake.

### Identification of resin sources–sampling of bees and trees

To identify the plant sources of resins collected by bees we compared the chemical composition of resin loads of individual foragers with resin collected from locally occurring tree species. Resin loads were sampled from returning foragers by capturing them in a plastic tube with a grid attached at one side (as commonly used to mark honeybee queens) and carefully removing resin loads from the corbicula of their hind legs using forceps. Tree resins were collected from individual trees within the close surrounding (approximately 0.5–2 km flight distance) of our study apiaries by gently scraping natural resin secretions of buds or, in case of *Picea abis* and *Pinus sylvestris*, from fresh wounds. Samples of three buds or multiple sprouts were pooled per tree, and, if possible, a minimum of three individuals per tree species were sampled and analyzed to account for variation between individuals. We collected resins from eight different tree species: *Aesculus hippocastanum* (3 samples/trees), *Alnus glutinosa* (3), *Betula alba* (4), *Populus balsamifera* (1), *Populus tremula* (3), *Populus x canadensis* (9), *Picea abis* (2) and *Pinus sylvestris* (3). Overall 37 samples of bees and 28 samples of trees were collected at seven different apiaries to account for possible variation between sites. All samples (of bees and trees) were immediately immersed in hexane and cooled (-18 C°) until chemical analysis. We additionally took samples of propolis from two individual colonies per site. Propolis was obtained by placing commercial plastic grids on top of the frames from June to September and collected once in September. For chemical analysis 0.3 g of ground propolis from each grid were extracted in hexane.

### Chemical analysis

Hexane extracts of propolis and resin samples were analyzed by gas chromatography coupled with mass spectrometry (GC-MS) using a Hewlett Packard HP 6890 Series Gas Chromatographic System coupled to a Hewlett Packard HP 5973 Mass Selective Detector (Agilent Technologies, Böblingen, Germany) and a Shimadzu QP2010 Ultra GC/MS (Shimadzu, Duisburg, Germany). The following tree resin samples, i.e. POL1 (poplar Px2), POL2 (poplar Px2), LG109 (spruce) and bee collected resins: LG111 (spruce), OL126 (orange), BB204 (whitish), OL131 (ocker), OL128 (orange), BB206 (brown), were analyzed by the Hewlett Packard HP 5973 Mass Selective Detector (Agilent Technologies, Böblingen, Germany), while all other samples were analyzed with Shimadzu QP2010 Ultra GC/MS (Shimadzu, Duisburg, Germany). The GCs were equipped with a J & W, DB-5 fused silica capillary column (30 m×0.25 mm ID; df = 0.25 μm; J & W, Folsom, CA, USA). Temperature was programmed from 60°C to 300°C with a 5°C/min heating rate and was held at 300°C for 10 min. Helium was used as carrier gas with a constant flow of 1 ml/min. Injection was carried out at 250°C in the splitless mode for 1 min. Electron impact mass spectra (EI-MS) were recorded at an ionization voltage of 70 eV and a source temperature of 230°C. We used the Windows version of the ChemStation software package (Agilent Technologies, Böblingen, Germany) and the GCMSsolution Vers. 2.7 for data acquisition. Sample chromatograms were analyzed manually by characterizing each peak of a chromatogram and comparing them across samples/chromatograms. Peaks were characterized by their retention times and mass spectra. A personal library was used to match peaks of different chromatograms, where we considered peaks with the same retention times and mass spectra the same substances (including both identified and unidentified substances). We then calculated relative peak areas for each compound by dividing the integrated area of each peak by the total area of all peaks. We additionally ran synthetic alkanes (Sigma-Aldrich, Munich, Germany) to confirm identification and to calculate Kovats indices. To finally identify different substance classes and, where possible, substances, if possible, we used three commercially available mass spectra libraries (Wiley 275, NIST 98 and Adams EO library 2205) and diagnostic ions and Kovats indices to verify our characterization.

### Statistical analysis

To analyze the effect of the surrounding habitat (site effects) on the spectrum of resin collected by individual colonies we performed permutation tests based on relative frequencies of different resin color types (Adonis command, R package vegan [47]). Differences in resin diversity were assessed by comparing resin type richness (pooled across colonies) between apiaries/sites using a Pearson’s chi^2^-test. Kruskal-Wallis ANOVA was used to investigate site-specific differences in the proportion of single resin types collected. Possible correlations between the intake of different resin types and between temperature and the total number of resin foragers were analyzed using Kendall's rank correlation tau test [48]. Prior to analyses data was checked for normality and homogeneity of variances using Shapiro-Wilk test and Fligner-Killeen test.

To identify the botanical sources of bee-collected resins we compared the chemical composition of bee-collected resins and tree resins. Prior to analysis trace compounds accounting for less than 0.05% (resin samples) or 0.1% (propolis samples) of the total peak area had been removed. Thresholds for resin and propolis samples were different to account for the higher concentration of propolis samples compared to bee and tree resin samples. All remaining (identified and unknown) substances were displayed by their relative peak areas (see above), which were used for all subsequent statistical analyses. We first investigated differences in the chemical composition of bee-collected resins between different color types. Second, we analyzed inter- and intra-specific differences in the chemical composition of tree resins. We then analyzed similarities of bee-collected resins and tree resins. All comparisons ((1) resin color types, (2) tree resins, (3) bee-collected resins/tree resins) were performed by permutation tests (Adonis command, R package vegan, 10000 runs) based on Bray-Curtis distances between compounds. Two-dimensional NMDS (non-metric dimensional scaling) based on Bray-Curtis distances [47], was used to generate ordination figures. Additionally, differences in the chemical composition of propolis samples from different colonies and different sites were analyzed as described above but excluding all alkanes and alkenes as potentially bee-/ wax-derived compounds. All analyses were performed in R statistical software version 3.4.1 [48]. Due to multiple testing the same data sets we only considered p-values < 0.01 significant.

## Results

### Resin intake

**Fig 2:**
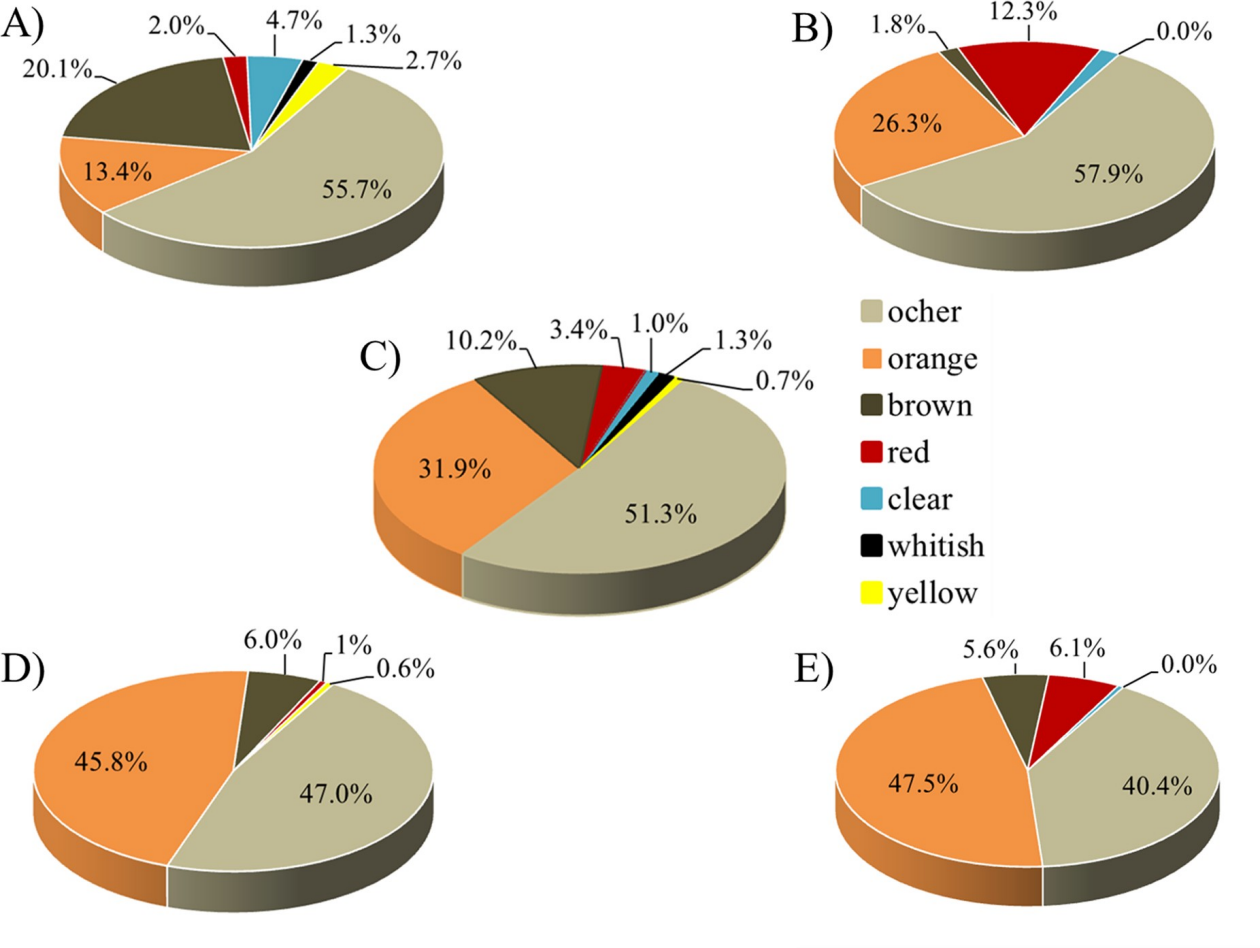
Percentages of different resins types collected by resin foragers from seven apiaries in Lower Saxony, Germany. C) total of foragers (N = 661), A)–E) four exemplary sites (A) site Lg (N = 149); B) site Ml (N = 57); D) site Gr (N = 168) and E) site Ol (N = 198)). For meaning of abbreviations and site descriptions see Table 1. Different resins types were characterized by color (i.e. “ocher”, “orange”, “brown”, “red”, “yellow” “whitish”, and “clear”) during field observations and subsequently verified by chemical analysis (via gas chromatography and mass spectrometry, GC-MS).

Overall, we observed 661 returning resin foragers from 28 colonies of seven apiaries located at seven different sites (Fig 2). The average number of resin foragers (calculated as the average total number of resin foragers observed across all colonies and sites for each month) was lowest in June (mean number of bees [± SD]: 4 ± 5), increased in July (6 ±5) and August (6 ± 6) and decreased again in September / October (5 ± 5). The total number of resin foragers further increased with increasing temperature (Kendall's rank correlation tau: *z* = 2.42, *P* < 0.015, *tau* = 0.16). Across colonies and apiaries, we categorized seven distinct resin types according to their color and chemical composition, i.e. „ocher”, orange-brown (type “orange”), red-brown (type “red”), green-brown (type “brown”), “yellow”, “clear” and “whitish”, with “ocher” (51% of all recorded resin foragers) and “orange” (32%) most frequently observed (Fig 2).

**Fig 3:**
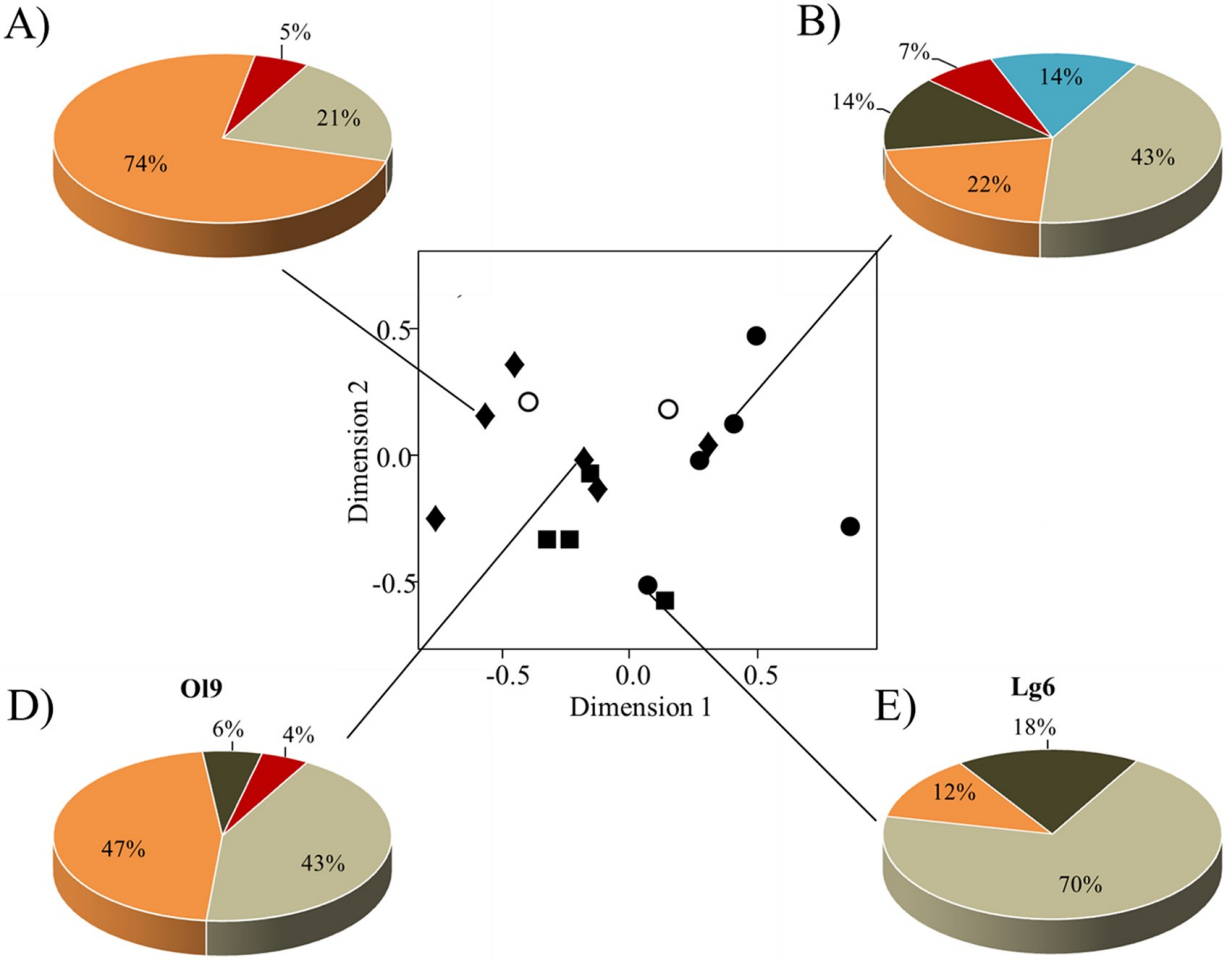
Variation in resin spectra collected by 16 colonies from four different apiaries/sites. Bees collected varying amounts of up to five different types of resin (“ocher”, “orange”, “brown”, “red”, “clear”). Ordination figure (C) shows similarities among colonies from different sites based on Bray-Curtis dissimilarities between samples (stress value = 0.06). Sites are represented by different symbols: closed circle, “Lg”; closed diamond, “Ol”; closed square, “Gr”; open circle, “Ml” with each symbol representing a single colony (for meaning of abbreviations see Table 1). Circular charts (A–B) and C—D) display proportions of resin types collected by four examplary colonies from two different sites (site “Ol”: A) colony ID 8: N resin samples = 19, and B) ID 9: N = 88; site “Lg”: D) ID 4: N = 14 and E) ID 6: N = 40).

Our study colonies collected varying amounts of three to five different resin types out of the seven we observed in total (Fig 3). The resin spectra collected differed only marginally between study sites (Adonis: *R*^*2*^ = 0.47, *P* = 0.047), but varied strongly between some (even neighboring) colonies (Fig 3). While “ocher” resin was collected by all colonies irrespective of the location (Kruskal-Wallis test: *H* = 3.62, *P* = 0.31, Figs 2 and 3A–3E), proportion of foragers collecting “orange” varied significantly between study sites (Kruskal-Wallis-test: type “orange”: *H* = 17.82, *P* = <0.009) and was negatively correlated with”brown” resin (Kendall's rank correlation tau: z = -3.05, *P* = 0.002, tau = -0.23). Other resin types were collected at comparatively low numbers and only at specific sites (Fig 3). Resin diversity (i.e. resin type richness) did not differ between apiaries/sites (Pearson's Chi-squared test: *x*^*2*^ = 1.12, *P* = 0.77).

### Chemical analysis—Identification of resin sources

#### Bee-collected resins

Overall, resin samples from 39 bees and 28 individual trees of at least eight distinct species from eight different sites were analyzed by GC-MS, comprising overall 846 different compounds, of which 116 were identified based on their retention and Kovats indices (S1 and S2 Tables).

**Fig 4:**
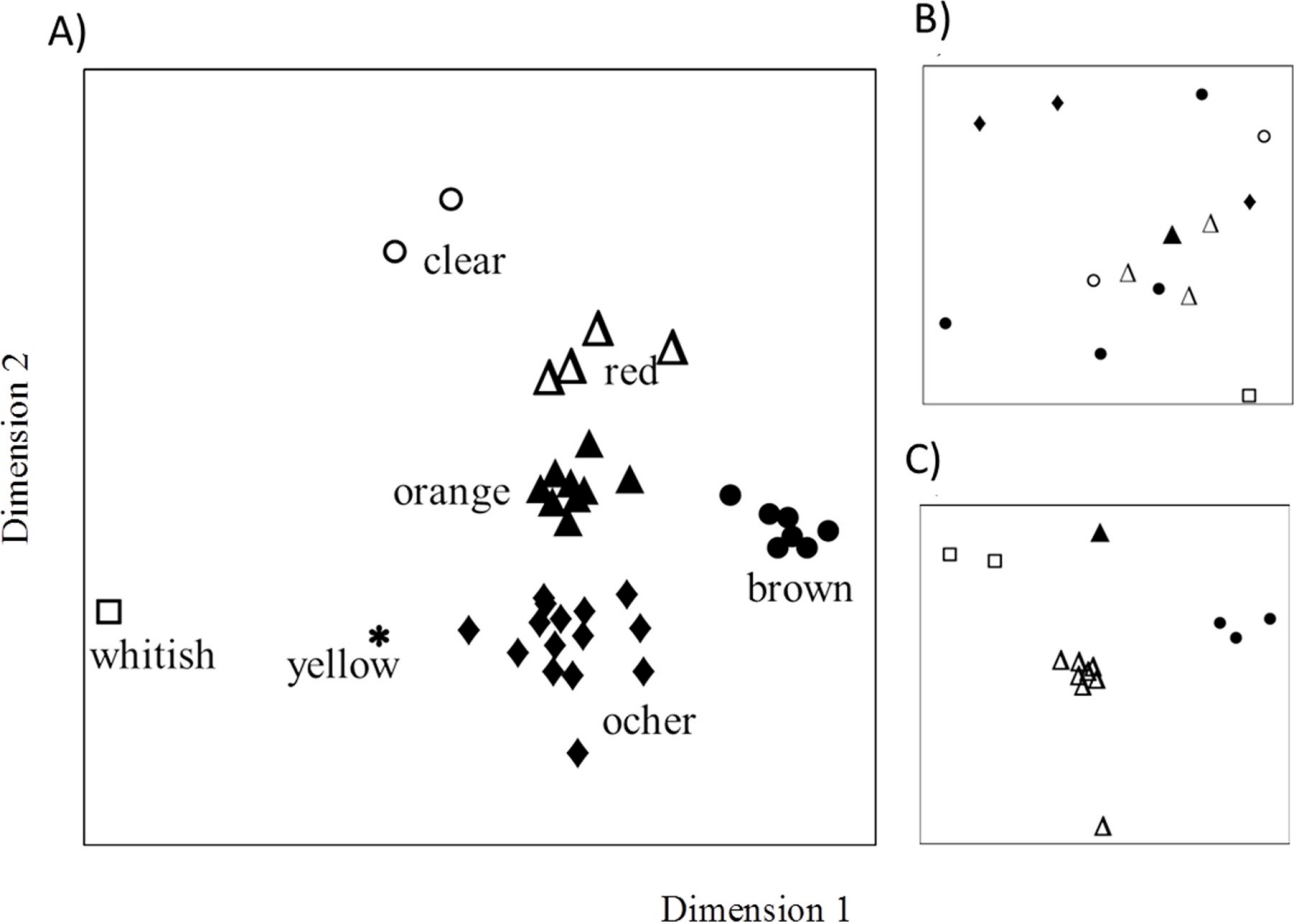
Chemical similarity within bee-collected resin samples. Chemical similarity of A) all resin samples collected by returning resin foragers (n = 39), B) only “ocher” samples (n = 14) and C) only “orange” and “red” samples (n = 15); ordination Figure from non-metrical three-dimensional scaling (NMDS) based on Bray-Curtis dissimilarities (stress value = 0.15). Different symbols represent different resin types (closed circle, “brown”; closed diamond, “ocher”; closed triangle, “orange”; open triangle, “red”; star, “yellow”; open circle, “clear”; open square, “whitish”) in A) and different sampling sites (closed circle, “Ez”; closed diamond, “Et”; closed triangle, “Lg”; open triangle, “Gr”; open circle, “Ml”; open square, “Ol”; for meaning of abbreviations see Table 1) in B) and C).

The chemical analysis of bee collected resins revealed seven distinct types (Adonis: *R*^*2*^ = 0.27, *P* = 0.001; Fig 4) correspondent to the visually assigned categories described above (Fig 4). The chemical composition of resin samples of the same type showed distinct patterns in the relative proportions of different substance classes (Table 2, Fig 4A), but also varied qualitatively and/or quantitatively within and between different apiaries/sites (Table 3, Figs 1, 4B and 4C). Variability within types was most pronounced within classes of terpenes, particularly sesquiterpenes.

**Table 2:** Prevalence of specific substance classes in different resin types.

Substance class	Orange		Ocher		Red		Brown		Clear		Yellow		Whitish	
	N	%	N	%	N	%	N	%	N	%	N	%	N	%
Aliphatic acids/ester	8 [3]	4 [7]	8 [6]	< 5	13 [0]	11 [2]	2 [2]	< 5	10 [1]	5 [0]	2	< 5	3	0
Phenolic acids/ester	6 [2]	2 [1]	4 [2]	12 [14]	9 [2]	8 [1]	2 [4]	< 5	3 [1]	< 5	0	0	0	0
Benzoic acid/ester	6 [1]	13 [9]	6 [3]	41 [9]	9 [1]	39 [5]	1 [1]	< 5	0	0	0	0	0	0
Phenolic compounds	5 [1]	< 5	6 [2]	5 [4]	5 [2]	< 5	3 [2]	< 5	7 [1]	< 5	3	< 5	4	< 5
Flavone/Flavonoids	5 [1]	10 [4]	1	< 5	6 [4]	8 [4]	3 [2]	< 5	1	0	0	0	0	0
Terpenoids	7 [1]	10 [7]	2 [1]	0	3 [1]	< 5	5 [14]	< 5	6 [0]	6 [1]	0	0	5	5
Monoterpenes/terpenoids	1	0	1	< 5	0	0	0	0	0	0	7	42	17	73
Sesquterpenes/terpenoids	18 [6]	40 [20]	17 [9]	27 [16]	6 [1]	< 5	33 [16]	77 [5]	0	0	2	9	3	0
Diterpenes/terpenoids	1	0	0	0	3 [0]	< 5	5 [3]	< 5	2 [0]	< 5	13	23	33	15
Triterpenes/terpenoids	0	0	2 [1]	0	2 [0]	< 5	2 [1]	< 5	22 [4]	37 [11]	0	0	0	0
Alcohols/aldehyds/ketons	5 [3]	< 5	3 [2]	< 5	7 [2]	< 5	4 [2]	< 5	10 [2]	< 5	1	0	0	0

Table shows mean [± standard deviation] number of compounds from specific substance classes and their mean relative proportions in the different resin types. The table refers to all compounds, which have been identified at least to substance class, i.e. 762 out of 846 compounds in total. For each type, the number of compounds (N) within a substance class was counted. The mean relative proportion of each substance class (%) in a specific resin type was calculated by summing up relative peak areas of all compounds of a specific substance class and dividing the sum by the number of samples containing this substance class.

Resin types “ocher” and “orange” varied strongly among samples of individual foragers from the same and different sites and showed partly little overlap among each other and with other resin types (Table 3, Fig 4B, S2 Fig). Both types were characterized by a large content of benzoic and phenolic acids as well as diverse sesquiterpenes and phenolic compounds (Table 2). In all samples of type “ocher” and “orange”, benzoic acid (m/z 77, 122; Kovats-Index: 1171) was the most abundant component, with type “ocher” comprising 15.9–47.7% (mean 32.1% of the relative peak area) and type “orange” 0.8–23.3% benzoic acid (mean: 12.1% of the relative peak area). “Ocher” and “orange” resin further shared one flavonoid component (retention time (RT) 39.0 min) and about six sesquiterpenes (e.g. *alpha*-humulene, E-isoeugenol, *alpha*-guaiene and caryophyllene/oxide). In contrast to “ocher” and “orange” resin, variability in chemical composition was relatively low among “brown” resin samples from different sites (Fig 4, Table 3). They contained mainly sesquiterpenes and other terpenoids, only few phenolic compounds and almost no benzoic or phenolic acids (Table 2). Three sesquiterpenes (Z-*beta*-farnesene, and two unidentified sesquiterpenes (retention time 21.58 min and 24.78 min) were most abundant (comprising 33.3–54.5%, mean: 46.4% of the total peak area). They occurred exclusively in all samples of type “brown” and were further found in all propolis samples. Interestingly, these sesquiterpenes where present only in resins of *B*. *alba* tree species that were used by bees but not in resins of species not collected by bees (Fig 4).

**Table 3:** Compositional variability among samples of the same resin types.

Chemotype	N shared samples^a^	N components^b^	Area overlap [%]^c^	Area overlap [%] per site^d^	N study sites	Matches with propolis^e^
Ocher	13	49 [20]	1.6	32.1 [9.6]	5	16 [5]
Orange	8	71 [17]	17.3	93.9 [4.6]	3	19 [2]
Red	3	80 [10]	78.8	78.8 [n.a.]^f^	1	16 [3]
Brown	7	67 [7]	62.9	92.5 [0.1]	4	15 [2]
Clear	2	79 [4]	67.6	n.a.	2	12 [2]
Whitish	1	74	n.a.	n.a.	1	0
Yellow	1	38	n.a.	n.a.	1	6 [1]

^a)^ Number of samples of this type.

^b)^ Mean number [± standard deviation] of total components.

^c)^ Mean proportional area overlap of all compounds shared among all samples of this type.

^d)^ Proportional area overlap [± standard deviation] of compounds shared by all samples of this type from a specific study site, the number of study sites where this type occurred (N study sites). Area overlap basically corresponds to compound overlap, but weighs more common compounds (large area) more than less common ones (small area).

^e)^ Number [± standard deviation] of compounds shared with propolis from the same apiary (matches with propolis for two propolis samples per site).

^f)^ n.a. = overlap was not calculated when there were less than two samples to compare.

#### Tree resins

**Fig 5:**
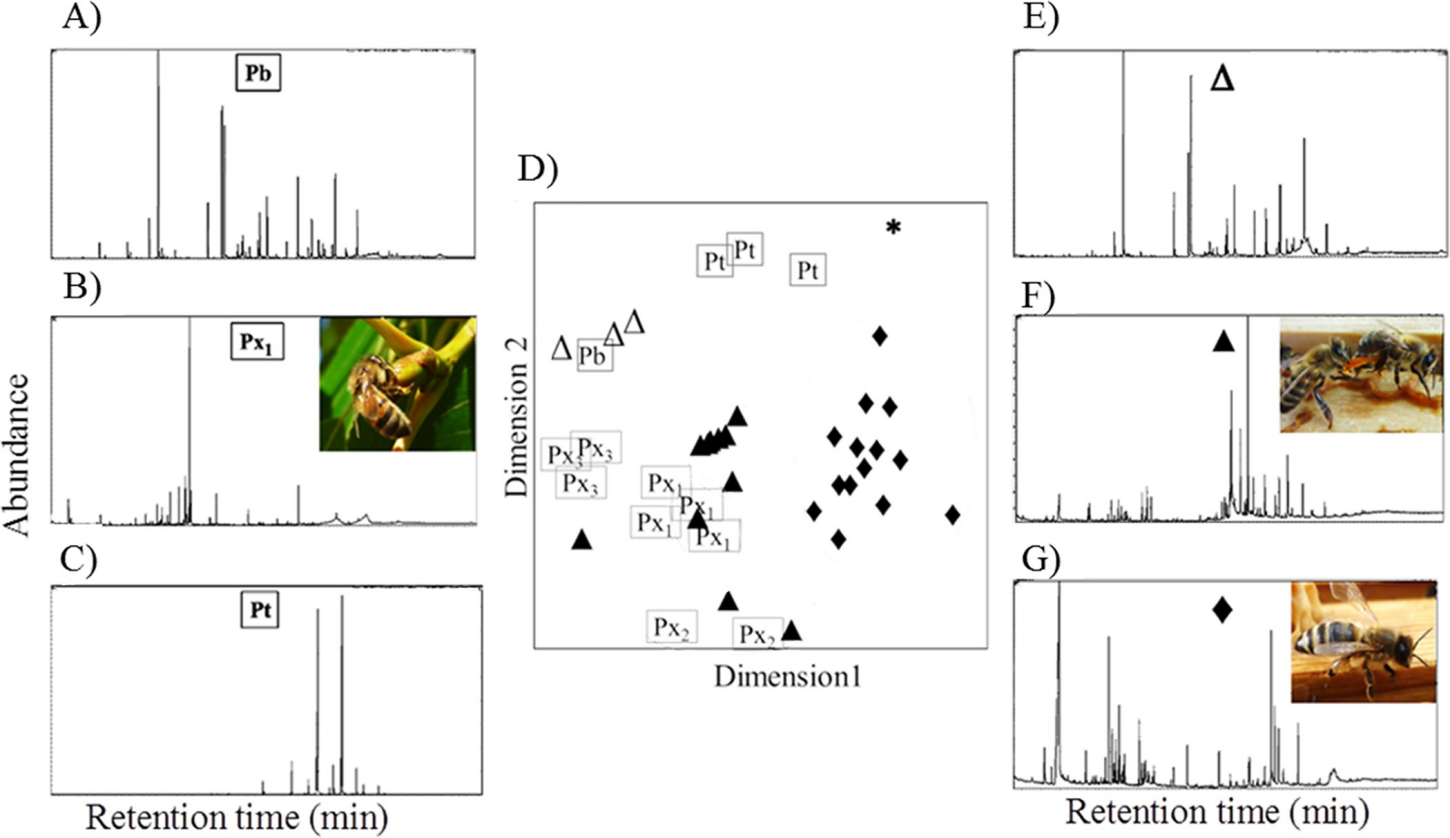
Similarity in chemical composition of tree bud resin from poplar tree species and bee-collected resins (“ocher”, “orange”, “red” and “yellow”). Bee-collected resins did not unambiguously correspond to any of the other sampled tree species. Ordination Figure (D) is based on Bray-Curtis dissimilarities between samples (stress value = 0.18). Different letters indicate different tree species: Pb: *Populus balsamifera*; Pt: *Populus tremula*; Px_1_-Px_3_: three different chemotypes of *Populus x canadensis*. Different symbols represent different resin types: closed diamond: “ocher”; closed triangle: “orange”; open triangle: “red”; star: “yellow”. A–C and E–G) Exemplary chromatograms of hexane extracts from tree buds (left) and bee-collected resins (right): A) *P*. *balsamifera*; B) *P*. *x canadensis* (chemotype Px_1_); C) *P*. *tremula* (chemotype Px_3_); E) “red” bee-collected resin; F) “orange” bee-collected resin; G) “ocher” bee-collected resin; chromatograms display retention times in minutes on the x-axis and mass current (mc) on the y-axis; pictures in chromatograms show examples of resin foragers carrying the respective resin chemotype.

**Fig 6:**
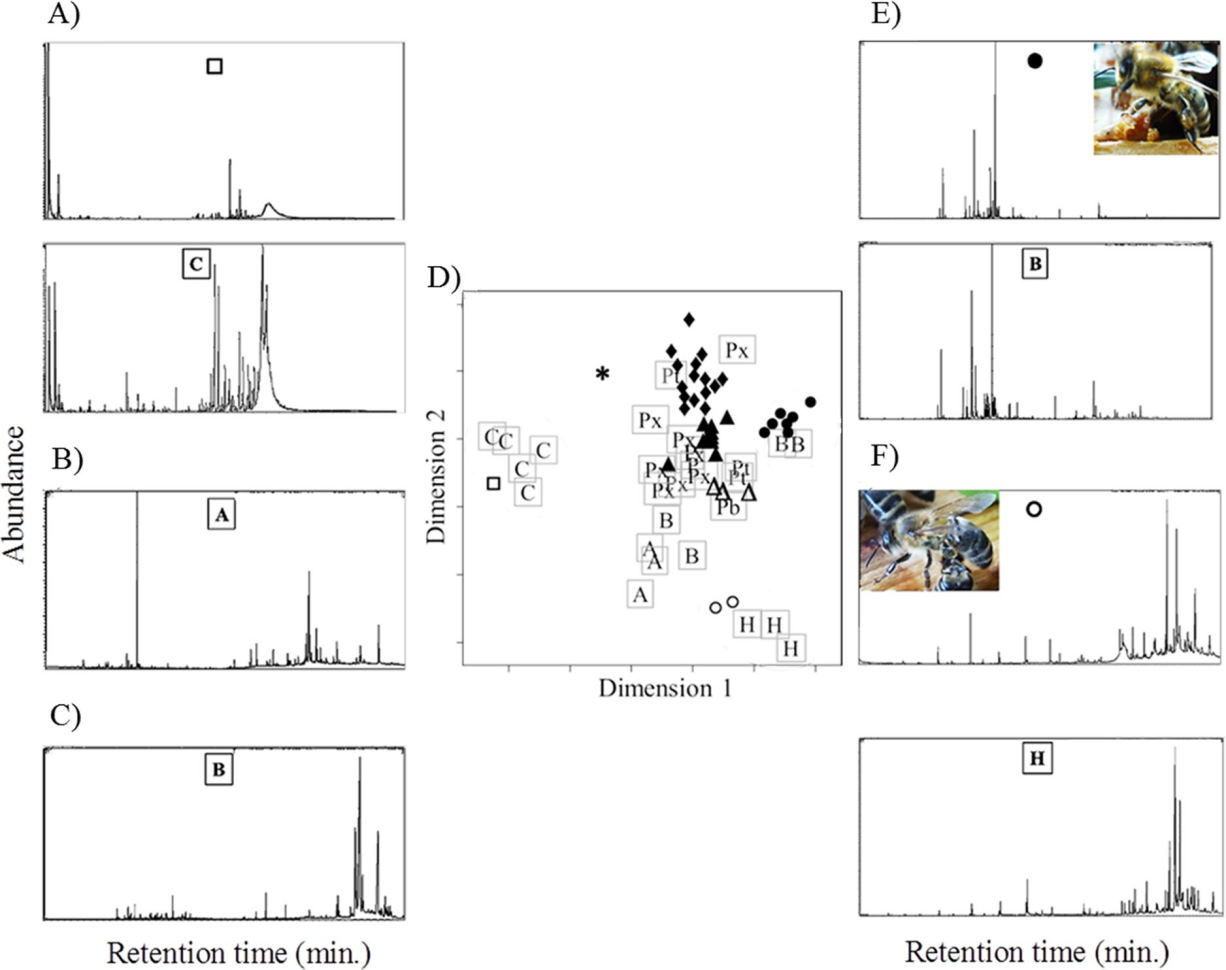
Similarity in chemical composition of tree bud resins and bee-collected resins. Tree bud resins (letters in ordination Figure; n = 30) and bee-collected resins (symbols; n = 39) from 17 colonies at seven different apiaries/sites. Ordination Figure (D) is based on Bray-Curtis dissimilarities between samples (stress value = 0.20). Different letters indicate different tree species: C, conifers (*Picea abis* / *Pinus sylvestris*); Pb, *Populus balsamifera*; Px, *Populus x canadensis*; Pt, *Populus tremula*; B, *Betula alba*; A, *Alnus glutinosa*; H, *Aesculus hippocastanum*. Different symbols represent different resin types: closed circle: “brown”; closed diamond: “ocher”; closed triangle: “orange”; open triangle: “red”; star: “yellow”; open circle: “clear”; open square: “whitish”. A)—F) Exemplary chromatograms of hexane extracts from tree bud resins and bee-collected resins: A) top: corbicula resin “whitish”, bottom: *P*. *abis*; B) *A*. *glutinosa*; C) *B*. *alba*; E) top: corbicula resin “brown”, bottom: *B*. *alba*; F) top: corbicula resin “clear”, bottom: *A hippocastanum*; chromatograms display retention times in minutes on the x-axis and mass current (mc) on the y-axis; pictures in chromatograms show examples of resin foragers carrying the respective resin type.

Tree resins were chemically highly diverse with up to 300 and more different compounds (including trace compounds) in a single sample (mean number of compounds per sample [± standard deviation] = 230 ± 133 compounds). Highest diversity was found for bud resins of *P*. *x canadensis* (Fig 5D). The chemical composition varied between different tree species (Adonis: *R*^*2*^ = 0.60, *P* = 0.009; Fig 6D). Poplar displayed the comparatively greatest intra-specific variability (Fig 6C, 6D and 6E and Fig 5A–5D). Chemical composition further varied between *P*. *x canadensis* individuals from different sites, but also between neighboring trees (Fig 5D), displaying three distinct *P*. *x canadensis* (chemo)types (henceforth referred to as *P*. *x canadensis* type1, type2 and type3) of which two were similar to the resin type “orange” (Fig 5D). Resin samples of *P*. *x canadensis* type1 and type2 as well as *P*. *balsamifera* were characterized by comparatively large amounts of benzoic and phenolic acids (proportion of the total peak area [± standard deviation]: 26.3 ± 14.3%) and low quantities of monoterpenes (proportion: 0. ± 0.3), whereas samples of *P*. *x canadensis* type3 showed the opposite trend (proportion of benzoic and phenolic acids: 0.2 ± 0.2; proportion of monoterpenes: 3.9 ± 1.3). For *B*. *alba*, we found two different resin (chemo)types (each with two individuals) at overall three different sites, with two neighboring individuals from the same site belonging to different (chemo)types (Fig 6D).

The following bee-collected resin types chemically matched specific tree species: type “orange” chemically matched *P*. *x canadensis* type1 and type2, type “brown” matched *B*. *alba* type1, type “red” matched *P*. *balsamifera*, type “clear” matched *A*. *hippocastanum* and type “whitish” matched the two sampled conifers (*Picea abis* / *Pinus sylvestris*). No clear matches were found for types “ocher” and “yellow” (Fig 5). Thus, honeybees collected resins from tree buds of *P*. *x canadensis*, *P*. *balsamifera*, *B*. *alba*, *A*. *hippocastanum* and from wounds of coniferous tree species (e.g. *P*. *abis* or *P*. *sylvestris*) (Fig 6). None of the bee collected resins showed chemically similarity to resin samples collected from *A*. *glutinosa*, *B*. *alba* type2 and *P*. *x canadensis* type3 (Fig6). While resin type “ocher” did not unambiguously correspond to any tree resin, it contained up to nine compounds that were exclusively found in poplar resin samples. Likewise, resin type “yellow” showed some overlap with type “ocher”, but further comprised compounds (e.g. D-limonene and 3-carene) which were primarily found in coniferous resins.

**Fig 7:**
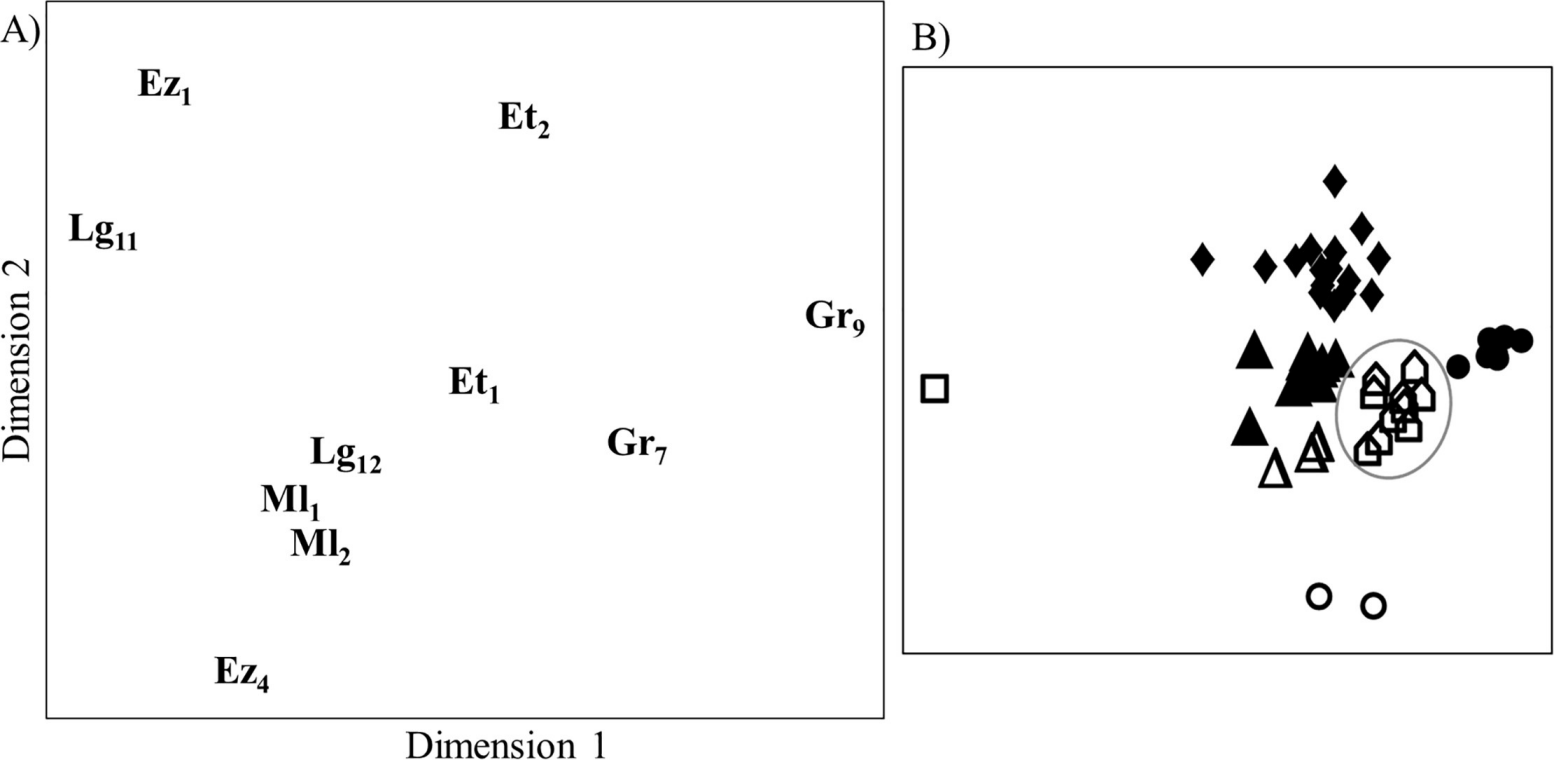
**Similarity in chemical composition of propolis samples from 10 colonies of five different apiaries/sites (A) and propolis samples in relation to bee-collected resin types (B).** Letters indicate different apiaries/sites (for meanings of abbreviations see Table 1), numbers mark different colonies. Ordination Figures are based on Bray-Curtis dissimilarities between samples (stress value = 0.07). Different symbols in B) represent different resin types: closed circle: “brown”; closed diamond: “ocher”; closed triangle: “orange”; open triangle: “red”; open circle: “clear”; open square: “whitish”.

Finally, propolis samples from 10 colonies of five apiaries differed in their chemical composition between colonies of both the same site and different apiaries (Fig 7). Compared to single bee-collected resins, propolis samples were more similar to each other than to any of the single resins types (Fig 7). Moreover, we found only relatively few compound matches between bee-collected resins and propolis samples (Table 2).

## Discussion

Honeybees collect plant resins for various purposes, such as defense against pests and pathogens and for nest construction and sealing [36,49]. Despite its importance, very little is known on precise resin sources as well as variation in the spectra and diversity of resins collected by honeybees.

### Resin sources used by honeybees

We chemically compared resin collected from resin foragers and resin sampled from tree buds. Our results reveal that bees from our study colonies collected resin not only from several poplar species (*P*. *balsamifera*, *P*. *x canadensis*), but also from birch (*B*. *alba*), horse chestnut (*A*. *hippocastanum*) and coniferous trees (either *P*. *abis* or *P*. *sylvestris*). While the use of poplar resins had already been confirmed by previous studies [39,45,50], our study provides first evidence for the exploitation of a broader spectrum of resin sources, including poplar species, birch, horse-chestnut and conifers.

However, despite clear compositional similarity, we hardly found close matches between bee-collected resins and plant samples, which may partly be explained by the high variability within and among different plant taxa. Intra-specific variability in resin chemistry was particularly pronounced among tree individuals of poplar and birch, both known to build a large variety of hybrids which are often difficult to distinguish based on morphological traits [51–63], but can strongly differ in resin chemical composition [39,62].

Interestingly, we could not unambiguously identify the precise plant source of the frequently collected “ocher” resin type. Although this type contained a few compounds (up to nine) also found in *P*. *x canadensis* and *P*. *tremula* resins, its chemical composition did not fully overlap with that of the sampled poplar bud resins. In fact, at least 45 compounds (mostly sesquiterpenes) detected in *P*. *x canadensis* resins were missing in the “ocher” resin type.

Missing or incomplete matches between bee-collected resins and bud resin samples of potential source plants may indicate that bees either collected from source trees or tree parts (e.g. crown) which we did not sample and which were chemically different from our tree samples. Alternatively, honeybee resin foragers may somehow alter resins during the collection process, e.g. by enzymatically degrading specific compounds or changing the composition of compounds typically produced by the tree.

Moreover, bee-collected samples of a specific resin type (e.g. “orange” and “brown”) from colonies of different apiaries were occasionally very similar (48.3–62.9% area overlap, see Table 3 and Figs 5 and 6), suggesting that bees select specific (chemo)types independent of location and recruit foragers to these plants [64]. In fact, bees did not necessarily collect resins from the closest sources available, but were highly selective and made distinct choices for specific (chemo)types even among closely related and partly neighboring tree species/individuals which strongly differed in their bud resin chemistry. Likewise, honeybees in North America collected resins from *P*. *deltoides* and *P*. *balsamifera*, but not from their numerous hybrids which were shown to produce bud resins with chemical profiles that were intermediate to the distinct profiles of their parental species [39].

### A functional role of benzoic acid and derivatives?

Both the “ocher” and “orange” resin type contained notable amounts of benzoic acid (i.e. 32.1 ± 9.6% in “ocher” and 12.1 ± 8.2% in “orange”), which was characteristic to all samples of both types. Benzoic acid also occurred in resins of some, but not all *P*. *x canadensis* and *P*. *tremula* individuals, although in much lower concentrations (i.e. 1.6 ± 2.4%). Benzoic acid is an aromatic compound commonly used for food preservation by humans due to its antimicrobial and antimycotic activity [65]. In plants, benzoic acids and their derivatives play a central role in attracting beneficial insects, e.g. pollinators, and in defense, e.g. against herbivores or other pests [66–68]. For example, in woody plants, increased production of benzoic acid in fruits takes place when they are infected with a fungus causing “tree cancer” (*Nectria galligena*) [69]. Consequently, benzoic acid has biological properties that may be beneficial to bees. Moreover, in contrast to most other chemical compounds commonly thought to be responsible for the biological activity of propolis (e.g. phenolic compounds or terpenes), benzoic acid is water-soluble. It is also regularly found in honey [70] and likely contributes to the preservability of honey. Given its prevalence in resin, benzoic acid in honey may actually be (at least partly) derived from resin storages (propolis). In fact, relative amounts of benzoic acid were much lower in propolis samples than in freshly gathered resin samples, suggesting that it evaporates in bee colonies (and e.g. into honey). Similarly, three other typical constituents of poplar bud resin (pinobanksin, pinobanksin 5-methyl ether, and pinocembrin, which induce detoxifying CYP9Q enzymes) were also found to be accumulated in honey [71]. Due to its volatility, benzoic acid may further help honeybees to locate resin sources and thus function as chemical cue. Relatively large amounts of another benzoic acid derivate, benzyl benzoate (Kovats Index 1762), were also found in some bee collected resins of types “red” and “ocher” as well as in *P*. *balsamifera* (S2 Table). It is used as pharmaceutical acaricide e.g. against scabis and could thus protect bees against varroa mites (*Varroa destructor*).

### May honeybees induce secretion of preferred resin types?

As defense responses in plants can generally lead to an accumulation of defensive compounds such as benzoic acid [72], the preferential collection of distinct chemotypes (e.g. rich in benzoic acid) by our study bees may even indicate that bees particularly prefer resins produced as defense against stressors. The plant’s defensive response may further increase overall resin secretion (e.g. in aspen which had comparatively low amounts of bud resin within the study region, personal observation), as known for birch and poplar when browsed by snowshoe hares [73–75]. Besides mechanical wounding, chemical substances (e.g. enzymes) from oral secretions and insect-associated microbes can trigger defense responses and thus induce or maintain the secretion and/or alter their chemical composition [76–79]. In fact, resin foragers occasionally injure vegetative parts (e.g. buds or leaves) of plants [36,38] which may induce/increase resin secretion. In doing so they may transfer microbial symbionts (associated with the bees’ saliva) or pathogenic microbes (see [80–82]) and thus potentially trigger the production of preferred resin chemotypes. If this hypothesis holds true, this may further explain the missing overlap in chemical similarity between our tree and bee-collected samples.

### Resin intake, variability and diversity

Our study honeybee colonies clearly preferred two chemically distinct resin types, “ocher” (unknown source, likely poplar) and “orange” (*P*. *x canadensis*), over all other sources. A strong preference for some resins over others agrees with previous studies [39,43]. However, most of our colonies collected three or more different resin types, indicating that bees targeted a variety of different resins. Collected resin spectra in turn varied between apiaries/sites and colonies, indicating colony-specific site-independent collection targets. However, propolis samples from our colonies were chemically more similar to each other than to the bee-collected resin types, suggesting that colonies compile specific resin blends from a variety of different resins. Different resins show different functional properties, e.g. repel different antagonists [83]. Compiling resin from different sources thus increases the diversity of potentially bio-active compounds and may consequently better protect colonies against a variety of enemies [83]. In fact, our chemical analysis revealed a striking compositional chemical diversity within and among different resin types/sources. Thus, in addition to collecting resins from a comparatively broad spectrum of different plant species (as also shown for stingless bees: [46]), bees may further benefit from the large chemical intra-specific variability as found in resin from specific plant species.

## Conclusion

Our data shows that honeybees collect a comparatively broad and highly variable spectrum of resin sources and make distinct choices by preferring some resins (e.g. specific poplar species and chemotypes) over others. This finding corresponds to observations made for their tropical relatives (Apidae: Meliponini) [23,84], and likely insures that bees can combat a variety of antagonists sensitive to different resin sources and/or compounds (see [83]). Notably, the environment in our study area is shaped by intensive human impact, resulting in an altered assemblage of available plant species. For example, formerly occurring poplar species, such as *Populus nigra*, are now extremely rare and most probably not available to any of our apiaries. Instead, several planted and natural occurring hybrids of *P*. *nigra* and other poplar species are available in our study region. In an unaltered, intact environment, the bee’s preferences may be shifted to other (or even to more diverse) resources to compose the resin blends that best meet their current needs. However, the high chemical variability found among resin (chemo)types collected by our study bees may alternatively indicate that bees target and benefit from chemical rather than tree species biodiversity.

Future studies should attempt to unravel the causes underlying the choices and variability in resin intake observed in honeybees. Such knowledge would not only provide new insight into self-medication and external immunity in insects, but also provide important information for apicultural praxis.

## Supporting information

S1 Fig Location of the study apiaries. (DOCX)

S2 Fig Exemplary chromatograms. (DOCX)

S1 Table List of substance classes and number components identified from resin samples. (DOCX)

S2 Table List of all substances identified from resin samples of trees, bees and propolis and their relative amounts. (XLSX)
