# Supplementary material for: A clue on bee glue: New insight into the sources and factors driving resin intake in honeybees (Apis mellifera)
Source: PLoS One. 2019 Feb 6;14(2):e0210594. doi: 10.1371/journal.pone.0210594 (PMC6364881; doi:10.1371/journal.pone.0210594)
Supplement: S1 Fig — (DOCX) [file pone.0210594.s001.docx]

**Supporting information S1 Fig.**

**A clue on bee glue: New insight into the sources and factors driving resin intake in honeybees**


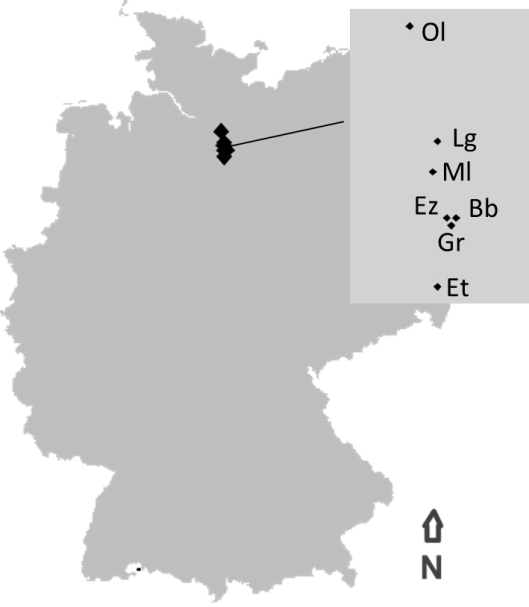


**S1 Fig.** **Location of the study apiaries**. Study apiaries were located at seven different sites in Lower Saxony, Germany. Distances among study sites are between 1.3 km (Ez and Gr) and 38 km (Ol and Et).
