# Supplementary material for: A clue on bee glue: New insight into the sources and factors driving resin intake in honeybees (Apis mellifera)
Source: PLoS One. 2019 Feb 6;14(2):e0210594. doi: 10.1371/journal.pone.0210594 (PMC6364881; doi:10.1371/journal.pone.0210594)
Supplement: S2 Fig — (DOCX) [file pone.0210594.s002.docx]

**Supporting information S2 Fig.**

**A clue on bee glue: New insight into the sources and factors driving resin intake in honeybees**

**S2 Fig. Exemplary chromatograms**

| **Ocher** | **Orange** |
| --- | --- |
|   1a) GR7-8.6.13 |   4a) GR9-6.5.13 |
|   1b) GR1-17.7.13 | 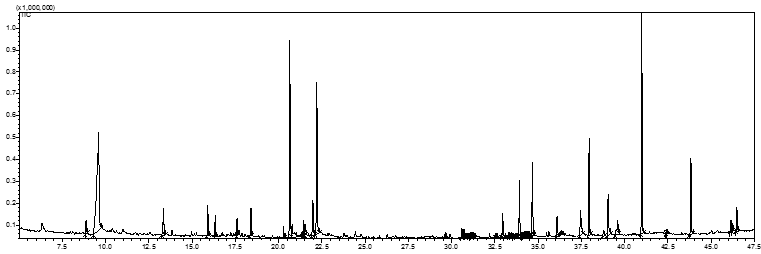  4b) GR9-8. 6.13 |
|   1c) GR3-16.8.13 |   4c) GR3-16.8.13 |
|   1d) ET5-12.7.13 |   4d) GR7-8.6.13 |
| 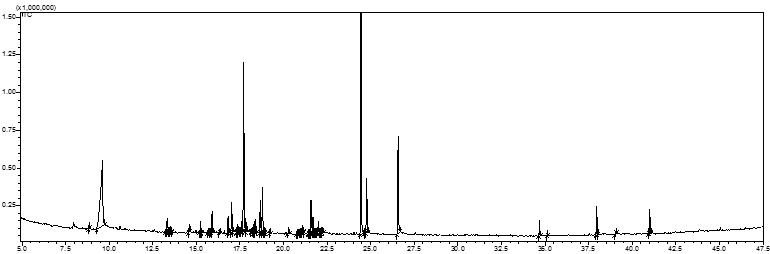  1e) ET5-12.7.13 |   4e) ET1-12.7.13 |
|   1f) LG6-16.7.13 |   4f) LG6-16.7.13 |

| **Red** | **Clear** |
| --- | --- |
|   2a) ML2-9.7.13 |   5a) ML4-2.10.13 |
|   2b) ML2-5.8.13 |   5b) GR1-3.10.13 |

| **Brown** | **Yellow** |
| --- | --- |

|   3a) GR3-8.6.13 |   6a) LG11-7.6.13  **Whitish** |
| --- | --- |
|  |  |
|   3b) GR5-3.10.13 | 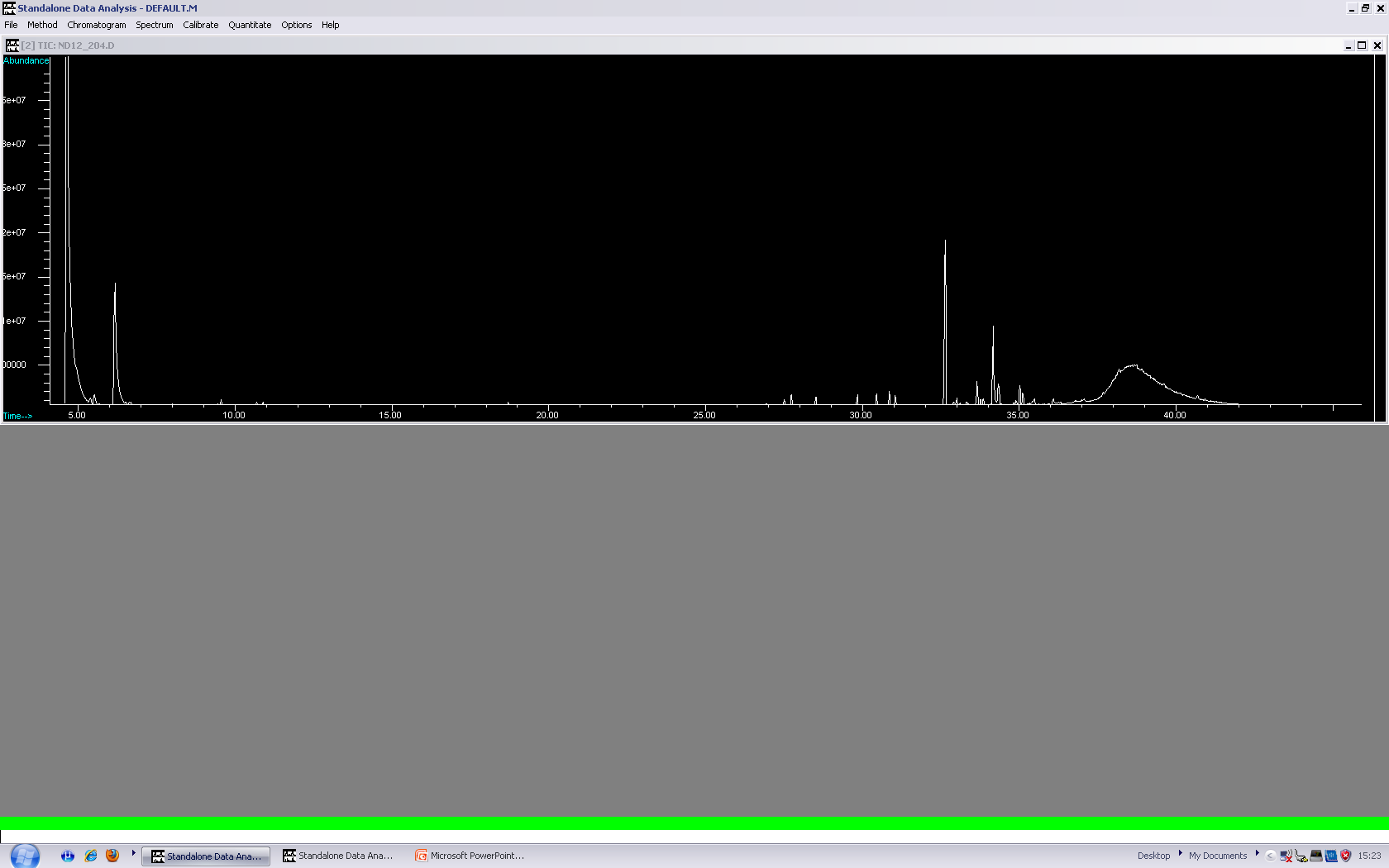  7a) BB2-3.9.12 |
|   3c) LG12-8.6.13 | **Brown**    3d) ET5-12.7.13 |

**S2 Fig.** **Exemplary chromatograms of hexane extracts from different bee-collected resins.** Chromatograms 1a) - 7a) represent different resin color types (orange, red, ocher, brown, yellow, clear, whitish) collected by single resin foragers between June and October 2013 at five different apiaries in Lower Saxony, Germany. Each peak represents a specific substance, where the peak area reflects the relative amount of a substance in a given sample. Capital letters give site name of the apiary (i.e. “Bb” Bienenbüttel, “Et” Ebstorf, “Gr” Grünewald, “Lg” Lüneburg, “Ml” Melbeck) with subsequent numbers reflecting colony IDs and sampling date. 1a) – f) resin type ocher (a) GR7-8.6.13, b) GR1-17.7.13, c) GR3-16.8.1, d) ET5-12.7.1, e) ET5-12.7.13, f) LG6-16.7.13); 2a) – b) type red (a) ML2-9.7.13, b) ML2-5.8.13); 3a) - d) type brown (a) GR3-8.6.13, b) GR5-3.10.13, c) LG12-8.6.13, d) ET5-12.7.13); 4a) – f) type orange (a) GR9-6.5.13, b) GR9-8. 6.13, c) GR3-16.8.13, d) GR7-8.6.13, e) ET1-12.7.13, f) LG6-16.7.13); 5a) – b) type clear (a) ML4-2.10.13, b) GR1-3.10.13); 6a) type yellow (a) LG11-7.6.13); 7a) type whitish (a) BB2-3.9.12). Resin samples were obtained by trapping returning resin foragers and removing their resin load from the corbicula of their hind legs with forceps. Hexane extracts of resin samples were analyzed by gas chromatography coupled with mass spectrometry (GC-MS).
