# Supplementary material for: A clue on bee glue: New insight into the sources and factors driving resin intake in honeybees (Apis mellifera)
Source: PLoS One. 2019 Feb 6;14(2):e0210594. doi: 10.1371/journal.pone.0210594 (PMC6364881; doi:10.1371/journal.pone.0210594)
Supplement: S1 Table — (DOCX) [file pone.0210594.s003.docx]

**Supporting information S1 Table**

**A clue on bee glue: New insight into the sources and factors driving resin intake in honeybees**

**S1 Table. List of substance classes and the respective numbers of compounds (Total N) identified from resin samples of tree buds, returning honeybee (*Apis mellifera*) foragers and from propolis samples.**

| **Substance Classes** | | **Total N** | **Constituents** **identified** | **Ret. time** | **KI** | **Tree species** | **N bees** | **N propolis** |
| --- | --- | --- | --- | --- | --- | --- | --- | --- |
| Acids and derivatives | | 167 |  |  |  |  |  |  |
| - Aliphatic acids /ester | | | Hexanoic acid | 4.88 | 967 | Px1 | 2 | 0 |
| (total:97) | | | Hexadecanoic acid methyl ester | 27.68 | 1927 | Px3 | 5 | 0 |
|  | |  | Linoleic acid | 31.47 | 2123 | Px2 | 6 | 0 |
| - Phenolic acids /ester | | | Benzoic acid | 9.69 | 1171 | Px1;Px2; Pt | 23 | 10 |
| (total:70) | | | Methyl salicylate | 10.18 | 1190 | Px1; Pb | 7 | 1 |
|  | | | benzyl benzoate | 24.33 | 1762 | Pb; Pt | 21 | 10 |
|  | |  | 2-Ethylhexyl salicylate | 25.13 | 1807 | B2 | 1 | 0 |
|  | |  | Benzyl salicylate | 26.43 | 1864 | Px2; Pb | 15 | 9 |
| Alcohols /aldehydes / ketones | | 53 |  |  |  |  |  |  |
|  | |  | Benzyl alcohol | 6.26 | 1026 | Px1;Pb; Pt | 17 | 1 |
|  | |  | Myristaldehyde (Tetradecanal) | 20.92 | 1611 | H | 3 | 0 |
|  | |  | Vanillin (4-Hydroxy-3 methoxybenzaldehyde) | 15.59 | 1393 | Pt | 12 | 3 |
| Other aliphatic compounds | | 73 |  |  |  |  |  |  |
| Phenolic compounds / phenylpropanoids | | 70 |  |  |  |  |  |  |
|  | |  | Chrysin | 40.25 | 2649 | - | 0 | 3 |
|  | |  | Coumaran (2,3-dihydro-Benzofuran) | 10.85 | 1219 | - | 1 | 7 |
| **S1 Table continued** | | | | | | | | |
| **Compound Classes** | | **Total N** | **Constituents identified** | **Ret. time** | **KI** | **Tree species** | **N bees** | **N propolis** |
| Terpenes / terpenoids | | 398 |  |  |  |  |  |  |
| - Monoterpenes and terpenoids | | | *alpha*-Pinene | 4.24 | 932 | Px1;Px3;C | 2 | 0 |
| (total:24) | |  | *beta*-Pinene | 5.08 | 974 | Px1;C | 2 | 0 |
|  | |  | *beta*-Myrcene | 5.28 | 988 | Px1;C | 2 | 0 |
|  | |  | 3-Carene | 5.74 | 1008 | Px3;C | 2 | 0 |
|  | |  | D-Limonene | 6.19 | 1031 | C | 2 | 0 |
|  | |  | *beta*.-Phellandrene | 6.23 | 1025 | C | 1 | 0 |
|  | |  | Eucalyptol | 6.24 | 1033 | Px3;C | 1 | 0 |
|  | |  | *trans*-*beta*-Ocimene | 6.56 | 1044 | Px3;C | 0 | 0 |
|  | |  | *gamma*-Terpinene | 6.87 | 1054 | Px3;Pt;C | 0 | 0 |
|  | |  | Terpinolene | 7.54 | 1086 | C | 1 | 0 |
|  | |  | *beta*-Linalool | 7.90 | 1095 | Px1 | 7 | 0 |
|  | |  | *alpha*-Campholene aldehyd | 8.51 | 1125 | - | 1 | 0 |
|  | |  | Sabinol | 8.90 | 1137 | C | 1 | 0 |
|  | |  | *trans*-Verbenol | 9.00 | 1140 | C | 1 | 0 |
|  | |  | Terpinen-4-ol | 9.99 | 1174 | Px3 | 1 | 0 |
|  | |  | L-*alpha*-Terpineol | 10.23 | 1186 | - | 1 | 0 |
|  | |  | Estragole | 10.43 | 1195 | C | 0 | 0 |
|  | |  | Bornyl acetate | 12.78 | 1282 | C | 1 | 0 |
|  | |  | Geraniol | 11.84 | 1249 | Px1 | 0 | 0 |
|  | |  | Thymol | 12.93 | 1289 | - | 1 | 5 |
|  | |  | Eugenol | 14.51 | 1356 | Px3 | 12 | 1 |
|  | |  | E-Isoeugenol | 16.97 | 1447 | Px2 | 15 | 2 |
| - Sesquiterpenes / terpenoids | | | *gamma*-Elemene | 14.04 | 1335 | A | 0 | 0 |
| (total:176) |  | | *alpha*-Cubebene | 14.42 | 1345 | Px1;Pt;A | 1 | 0 |
|  |  | | *alpha*-Ylangene | 14.98 | 1373 | Px1;Px3;Pt;B | 2 |  |
|  |  | | Copaene | 15.16 | 1376 | Px1;Px2;Pt;B;A | 5 | 0 |
|  |  | | *beta*-Bourbonene | 15.25 | 1384 | B;A;C | 1 | 0 |
| **S1 Table continued** | | | | | | | | |
| **Compound Classes** | **Total N** | | **Constituents identified** | **Ret. time** | **KI** | **Tree species** | **N bees** | **N propolis** |
|  |  | | Caryophyllene | 15.93 | 1408 | B | 1 | 0 |
|  |  | | Longifolene | 16.03 | 1407 | B | 0 | 0 |
|  |  | | *beta*-Ylangene | 16.15 | 1419 | Px1;B;A | 0 | 0 |
|  |  | | Caryophyllene <E> | 16.27 | 1417 | Px1;Px2;Px3;Pt; B;C | 28 | 1 |
|  |  | | *alpha*.-Bergamotene | 16.03 | 1411 | Px2 | 1 | 0 |
|  |  | | *beta*-Copaene | 16.53 | 1430 | Px1;B;A | 1 | 0 |
|  |  | | *alpha*-Guaiene | 16.65 | 1437 | Px3 | 18 | 0 |
|  |  | | *trans*-*alpha*-Bergamotene | 16.53 | 1432 | Px2 | 2 | 0 |
|  |  | | Z-*beta*-Farnesene | 16.77 | 1443 | B | 10 | 5 |
|  |  | | *alpha*-Humulene | 17.17 | 1452 | Px1;Px2;Px3;Pt; B;C | 25 | 0 |
|  |  | | Allo-aromadendrene | 17.29 | 1461 | Px1;Pt;B | 4 | 0 |
|  |  | | *gamma*-Muurolene | 17.66 | 1477 | Px1;Px3;Pt;B;C | 2 | 0 |
|  |  | | Ar-Curcumene | 17.73 | 1479 | Px2;C | 2 | 0 |
|  |  | | Germacene D | 17.79 | 1480 | Px3;A;C | 1 | 0 |
|  |  | | *beta*-Selinene | 18.09 | 1485 | - | 2 | 0 |
|  |  | | *alpha*-Selinene | 18.17 | 1498 | Px1;Px2;Px3;Pb | 18 | 0 |
|  |  | | *alpha*-Muurolene | 18.33 | 1500 | Px1 | 5 | 0 |
|  |  | | *alpha*-Bulnesene | 18.40 | 1505 | - | 8 | 0 |
|  |  | | *alpha*-Farnesene | 18.39 | 1508 | Pb | 4 | 0 |
|  |  | | *beta*-Bisabolene | 18.38 | 1509 | Px2 | 2 | 0 |
|  |  | | *gamma*-Cadinene | 18.60 | 1513 | Px1;Px2;Pb;B;A | 7 | 0 |
|  |  | | *trans*-calamenene | 18.84 | 1521 | Px1;Px3 | 1 | 0 |
|  |  | | *beta*-Sesquiphellandrene | 18.83 | 1521 | Px2 | 2 | 0 |
|  |  | | *delta*-Cadinene | 18.75 | 1522 | Px1;Px2;Px3;B; A;C | 8 | 0 |

| **S1 Table continued** | | | | | | | |
| --- | --- | --- | --- | --- | --- | --- | --- |
| **Compound Classes** | **Total N** | **Constituents identified** | **Ret. time** | **KI** | **Tree species** | **N bees** | **N propolis** |
|  |  | *alpha*-Copaene-11-ol | 19.12 | 1539 | Px2;Px3 | 2 | 0 |
|  |  | *alpha*-Calacorene | 19.27 | 1544 | Px1;Px3 | 1 | 0 |
|  |  | Selina-3,7(11)-diene | 19.28 | 1545 | Px3 | 0 | 0 |
|  |  | Elemol | 19.56 | 1548 | Px3 | 0 | 0 |
|  |  | Germacene D-4-ol | 20.11 | 1574 | B2;C | 0 | 0 |
|  |  | Caryophyllene oxide | 20.24 | 1582 | Px1;Px2;Px3;B;C | 30 | 7 |
|  |  | Gleenol | 20.43 | 1586 | Px1 | 0 | 0 |
|  |  | *gamma*-Eudesmol | 21.42 | 1630 | Px2;Px3 | 4 | 1 |
|  |  | alpha-Acorenol | 21.45 | 1632 | Px2;Px3 | 6 | 0 |
|  |  | Hinesol | 21.58 | 1640 | Px3 | 5 | 0 |
|  |  | T-Cadinol | 21.70 | 1638 | Px1 | 4 | 1 |
|  |  | *delta*-Cadinol | 21.74 | 1644 | Px1 | 4 | 0 |
|  |  | T-Muurolol | 21.74 | 1640 | Px1 | 4 | 0 |
|  |  | *alpha*-Cadinol | 21.94 | 1652 | Px1;C | 8 | 3 |
|  |  | Eudesmol | 22.01 | 1652 | Px2;Px3 | 8 | 1 |
|  |  | Bulnesol | 22.17 | 1666 | Px3 | 8 | 1 |
|  |  | Geranyl linalool<(Z,E)> | 27.21 | 1898 | C | 0 | 0 |
| - Diterpenes/ terpenoids | | Rosa-5,15-diene | 27.73 | 1933 | C | 1 | 0 |
| (total:94) |  | Cembrene | 28.06 | 1942 | C | 2 | 0 |
|  |  | Kaur-15-ene | 29.06 | 1997 | C | 1 | 0 |
|  |  | Geranyl linalool <(E,E)> | 29.59 | 2026 | C | 0 | 0 |
|  |  | 13-Epi-Manool | 30.12 | 2059 | C | 0 | 0 |
| - Triterpenes/ terpenoids (total: 67) | |  |  |  |  |  |  |
| - other terpenoids (total: 37) | |  |  |  |  |  |  |
| Unknown | 85 |  |  |  |  |  |  |
| **Total number** | **846** |  |  |  |  |  |  |

**S1 Table**. Individual components are displayed with their retention time (Ret. Time),Kovats retention index (KI) and occurrence in different tree species, bee-collected resins and propolis samples (given are numbers (N) of bee and propolis samples containing compound). Tree species as follows: Pb, *Populus balsamifera*; Pt, *Populus tremula*; Px1 - Px3, three different (chemo)types of unknown *Populus xcanadensis* hybrids; B – B2, two different (chemo)types of unknown *Betula alba* hybrids; A, *Alnus glutinosa*; C, conifers (*Picea abis* / *Pinus sylvestris*); H, *Aesculus hippocastanum*.
